# Correlation of IDH1 Mutation with Clinicopathologic Factors and Prognosis in Primary Glioblastoma: A Report of 118 Patients from China

**DOI:** 10.1371/journal.pone.0030339

**Published:** 2012-01-23

**Authors:** Wei Yan, Wei Zhang, Gan You, Zhaoshi Bao, Yongzhi Wang, Yanwei Liu, Chunsheng Kang, Yongping You, Lei Wang, Tao Jiang

**Affiliations:** 1 Department of Neurosurgery, Capital Medical University, Beijing Tiantan Hospital, Beijing, China; 2 Department of Neurosurgery, Nanjing Medical University, The First Affiliated Hospital, Nanjing, China; 3 Laboratory of Neuro-oncology, Tianjin Neurological Institute, Tianjin, China; Dartmouth College, United States of America

## Abstract

It has been reported that IDH1 (IDH1R132) mutation was a frequent genomic alteration in grade II and grade III glial tumors but rare in primary glioblastoma (pGBM). To elucidate the frequency of IDH1 mutation and its clinical significance in Chinese patients with pGBM, one hundred eighteen pGBMs were assessed by pyro-sequencing for IDH1 mutation status, and the results were correlated with clinical characteristics and molecular pathological factors. IDH1 mutations were detected in 19/118 pGBM cases (16.1%). Younger age, methylated MGMT promoter, high expression of mutant P53 protein, low expression of Ki-67 or EGFR protein were significantly correlated with IDH1 mutation status. Most notably, we identified pGBM cases with IDH1 mutation were mainly involved in the frontal lobe when compared with those with wild-type IDH1. In addition, Kaplan-Meier survival analysis revealed a highly significant association between IDH1 mutation and a better clinical outcome (p = 0.026 for progression-free survival; p = 0.029 for overall survival). However, in our further multivariable regression analysis, the independent prognostic effect of IDH1 mutation is limited when considering age, preoperative KPS score, extent of resection, TMZ chemotherapy, and Ki-67 protein expression levels, which might narrow its prognostic power in Chinese population in the future.

## Introduction

Primary glioblastoma (pGBM) is highly malignant and the most common type of primary brain tumors in adults. Regardless of surgery combined with radiation therapy and chemotherapy, median survival for pGBM patients ranges from 12–15 months after GBM diagnosis [1]. Thus new avenues have to be taken to discover effective strategies, requiring more insights into aberrant molecular mechanisms relevant to tumor biology and treatment [2]. Over the last decades, some of the characteristic genomic alterations have been reported to be associated with the origin and development of glioblastomas [3]. However, to date, only MGMT promoter methylation status has been demonstrated to be of clinical significance in prospective clinical trials in GBM patients [4]. Recent studies suggest that IDH1 R132 mutations are present in the majority of common adult gliomas but only occur in small fraction of pGBMs, and patients with IDH1 mutation have a better outcome than those with wild-type IDH1 in gliomas [5]. Further studies across the world have validated the exciting discovery [6], [7]. And it has been reported that patients with IDH1 mutation were also sensitive to Temozolomide (TMZ) in low-grade gliomas [8]. Consequentially, this raises questions regarding the capacity of IDH1 mutation for use as a prognostic or predictive marker for customized treatment in glial tumors in the near future. However, the frequency of IDH1 mutation and its clinical significance in Chinese patients with pGBM has not been elucidated systematically.

In the present study, to underscore the potential role of IDH1 mutation in pGBM, 118 Chinese patients with pGBM were assessed by pyro-sequencing for IDH1 mutation status, and the results were correlated with clinical characteristics and molecular pathological factors. IDH1 mutations were detected in 19/118 cases (16.1%). It should be pointed out that pGBM cases with IDH1 mutation were mainly involved in the frontal lobe, and also associated with younger age, methylated MGMT promoter, high expression of mutant P53 protein, low expression of Ki-67 or EGFR protein. Further Kaplan-Meier and Cox-regression analyses also demonstrated that IDH1 mutation was a prognostic but not an independent prognostic factor in Chinese patients with pGBM.

## Materials and Methods

### Tumor samples

One hundred and eighteen patients with primary GBM from the department of Neurosurgery at Beijing Tiantan Hospital were included in this study. All the patients underwent surgical resection between January 2006 and December 2009, and subsequently received radiation therapy and alkylating agent-based chemotherapy. Tumor tissue samples were obtained by surgical resection before the treatment with radiation and chemotherapy. Resected specimens were quick-frozen in liquid nitrogen and kept at −80°C until nucleic acid extraction. This study was approved by the Ethics Committee of Beijing Tiantan Hospital and written informed consent was obtained from all patients. Primary GBM was defined by two neuropathologists according to Scherer [9]. Only samples with greater than 80% tumor cells were selected. Clinical details, including the patient's sex, age at the time of diagnosis, preoperative Karnofsky Performance Status (KPS) score, tumor location, extent of resection, adjuvant chemotherapy, and the recorded date of disease progression or death were all noted.

### DNA pyro-sequencing for IDH1 mutation

Genomic DNA was isolated from frozen tumor tissues by using the QIAamp DNA Mini Kit (Qiagen). The genomic region spanning wild-type R132 of IDH1 was analyzed by Pyrophosphate sequencing using the following primers: 5′-GCTTGTGAGTGGATGGGTAAAAC-3′ and 5′-Biotin-TTGCCAACATGACTTACTTGATC-3′. The PCR analysis was performed in duplicate in 40 µl reaction volume, containing 1 µl of 10 µM each forward and reverse primer, 4 µl 10× buffer for, 3.2 µl of 2.5 mM dNTPs, 2.5 U hotstart Taq (Takara) and 2 µl of 10 µM DNA. The PCR conditions were as follows: 95°C–3 min; 50 cycles of 95°C–15 s, 56°C–20 s, 72°C–30 s; 72°C–5 min (ABI PCR system 9700). Single-stranded DNA was purified from the total PCR products and subjected to pyrosequencing on PyroMark Q96 ID System (QIAGEN) using the primer 5′- TGGATGGGTAAAACCT-3′ and EpiTect Bisulfite Kit (QIAGEN).

### DNA pyro-sequencing for MGMT promoter methylation

Bisulite modification of the DNA was performed using the EpiTect Kit (Qiagen). Two primers were used to amplify the MGMT promoter region: 5′- GTTTYGGATATGTTGGGATA -3′ and reverse: 5′-biotin-ACCCAAACACTCACCAAATC-3′. The PCR analysis was performed in duplicate in 40 µl reaction volume, containing 0.5 µl of 10 µM each primer, 4 µl 10× buffer, 3.2 µl of 2.5 mM dNTPs, 2.5 U hotstart Taq (Takara, Madison, WI) and 2 µl of 10 µM bisulphite-treated DNA. The PCR conditions were: 95°C–3 min; 40 cycles of 95°C–15 s, 52°C–30 s, 72°C–30 s; 72°C–5 min (ABI PCR system 9700). DNA was purified from the total PCR products using QIAamp DNA Mini Kit (Qiagen) and subjected to pyrosequencing (PyroMark Q96 ID System (Qiagen)) using the primer 5′-GGATATGTTGGGATAGT-3′ in accordance to the manufacturer's instructions. The methylation values obtained were averaged across the seven CpG loci tested within the MGMT promoter. The GBM samples were considered MGMT promoter methylated with an average methylation of >10%.

### Immunohistochemistry

Immunohistochemistry was performed as described as the previous report [10]. Briefly, surgical specimens were fixed in formalin, routinely processed and paraffin embedded. Five micron-thick sections were prepared, and immunohistochemical staining with streptavidin-biotin immunoperoxidase assay was performed using antibodies to Ki-67, MGMT, EGFR, VEGF, PTEN and mutant P53 (Santa Cruz Biotechnology, Santa Cruz, CA). The staining intensity was jointly scored by two pathologists without knowledge of clinical information on a scale of 0 to 3 (0, negative; 1, slight positive; 2, moderate positive; 3, intense positive). And scale of 0 and 1 and scale of 2 and 3 indicated low and high expression of the above proteins, respectively. Controls without primary antibody and positive control tissues were included in all experiments to ensure the quality of staining.

### Statistical analysis

Two clinical end-points were used to measure clinical outcome, progression-free survival (PFS) and overall survival (OS). PFS was defined as the time interval between the date of surgery and the date of first recurrence. OS was defined as the time interval between the date of surgery and the date of death. The survival function curve was calculated with the Kaplan-Meier method and the difference was analyzed using the two-sided log-rank test. Correlation of IDH1 mutation with clinicopathologic characteristics were evaluated by two-sided χ2 test or Student's t-test between the patient subgroups. Cox proportional hazard regression analyses were performed to assess the independent contribution of IDH1 mutation and clinicopathologic variables on survival prediction. All statistical analysis was performed in the SPSS 13.0 for Windows.

## Results

### IDH1 mutation in pGBM samples

**Figure 1:**
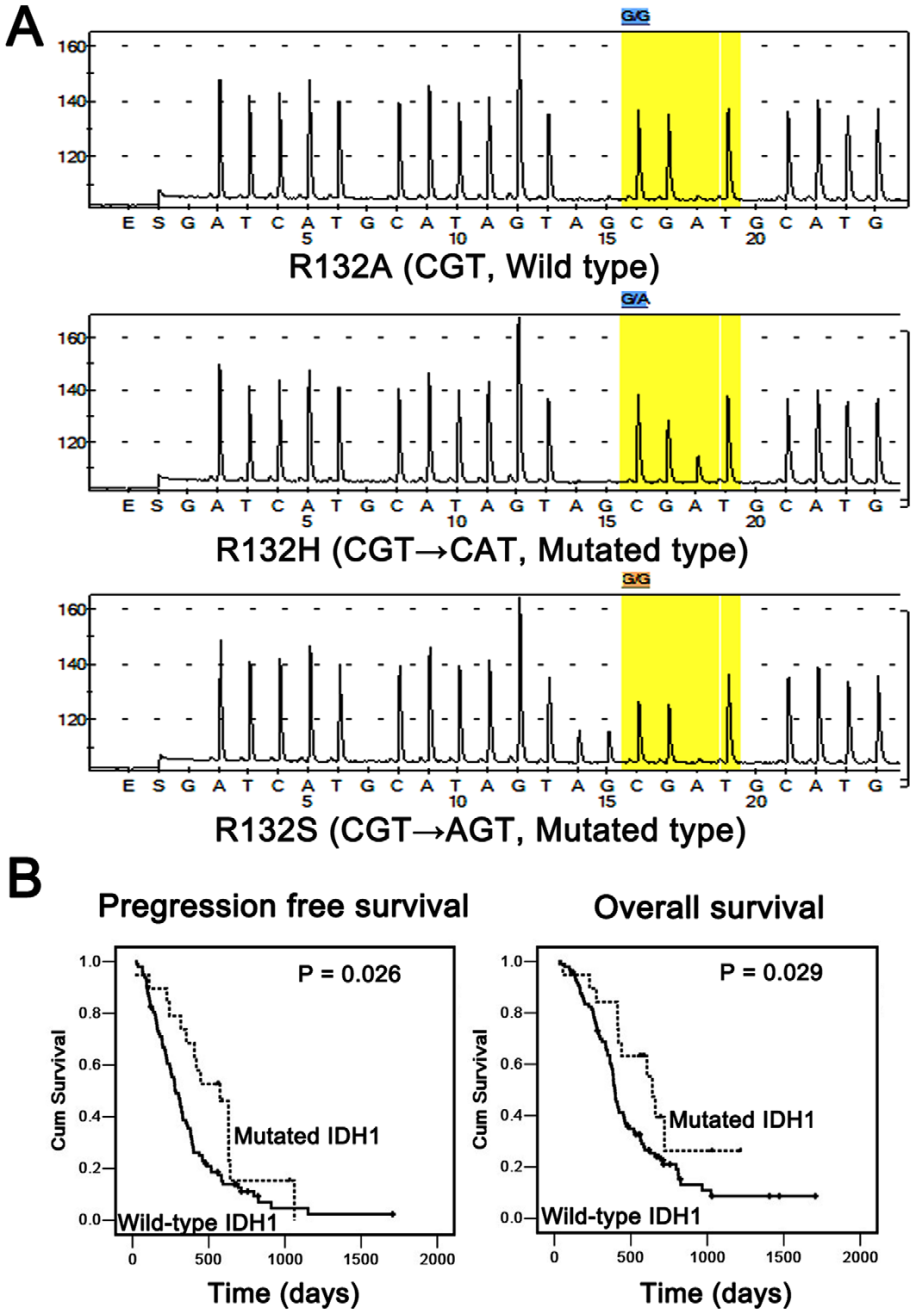
IDH1 mutations in pGBMs. All mutations by pyro-sequencing analysis were located at codon 132. And 89.5% of them were G395A transition (Arg→His), followed by C394A transversion (Arg→Ser; 10.5%) (A). Kaplan-Meier Survival Analysis showed that pGBM patients carrying an IDH1 mutation (dotted line) had significantly longer progression free survival (p = 0.026; log-rank test) and overall survival (p = 0.029; log-rank test) (B).

Of a total of 118 pGBM samples analyzed, 19 (16.1%) contained an IDH1 R132 mutation located at amino acid residue 132. And 89.5% of them were G395A transition (Arg→His), followed by C394A transversion (Arg→Ser; 10.5%) (Figure 1A). Patients with pGBM carrying IDH1 mutations were significantly younger than those without IDH1 mutations (mean age, 40.3 vs. 48.6 years; *p* = 0.002, t-test; Table 1). And 16/19 pGBM cases (84.2%) with IDH1 mutation were located in the frontal lobe, while only 36/99 (36.4%) with wild-type IDH1 were involved in frontal lobe (*p*<0.001, two-sided χ^2^ test; Table 1). The mean progression-free survival time of pGBM patients with IDH1 mutations was 497.0±267.8 days, significantly longer than that of patients without IDH1 mutations (342.3±261.6 days; *p* = 0.026, log-rank test; Figure 1B). And the mean overall survival time of pGBM patients with IDH1 mutations was 568.5±264.2 days, significantly longer than that of patients without IDH1 mutations (457.7±291.7 days; *p* = 0.029, log-rank test; Figure 1B).

**Table 1:** Clinical and molecular pathology features of pGBM samples in association with IDH1 mutations.

	IDH1 mutation	IDH1 wild type	*p* value*
**No. of cases**	19 (16.1%)	99 (83.9%)	
**Gender (Female/Male)**	8/11	36/63	0.796
**Age at diagnosis (year)**	40.3±9.3	48.6±13.2	**0.002** ‡
**MGMT promoter methylation (Unmethylated/Methylated)**	4/8	48/17	**0.015**
**MGMT (Low/High)**	8/11	38/58	1.000
**Ki-67 (Low/High)**	12/7	31/65	**0.018**
**EGFR (Low/High)**	10/9	22/74	**0.012**
**PTEN (Low/High)**	0/19	5/91	0.589
**VEGF (Low/High)**	5/7	17/35	0.737
**Mutant P53 (Low/High)**	1/18	30/66	**0.022**
**Tumor location (Location/Total)**			
**Frontal lobe**	16/19	36/99	**<0.001**
**Temporal lobe**	2/19	39/99	**0.017**
**Parietal lobe**	0/19	13/99	0.124
**Occipital lobe**	0/19	1/99	1.000
**Insula**	1/19	1/99	0.297
**Corpus callosum**	0/19	3/99	1.000
**Others**	0/19	6/99	0.588

*Two-sided χ test.

‡ Student's t-test.

### IDH1 mutation was associated with MGMT promoter methylation status, and Ki-67, EGFR, mutant P53 protein expression levels

**Figure 2:**
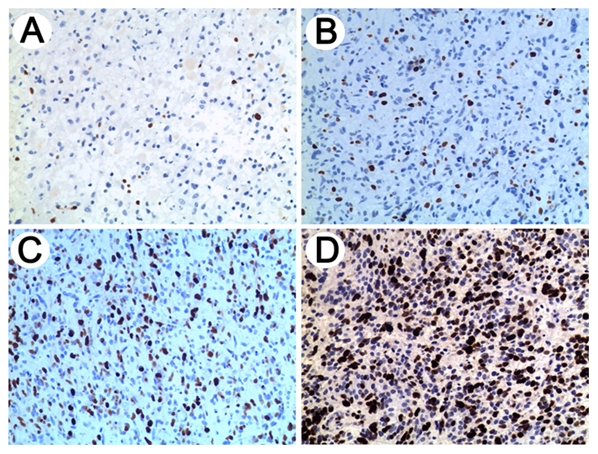
Representative antibody stainings for Ki-67. IHC results of Ki-67 are shown on a scale of 0 to 3 (0, negative (A); 1, slight positive (B); 2, moderate positive (C); 3, intense positive (D)). And scale of 0 and 1 and scale of 2 and 3 indicated low and high expression, respectively.

MGMT promoter methylation was assessed by pyrosequencing in 77 pGBMs. The Ki-67, MGMT, EGFR, PTEN and mutant P53 protein expressions were analysed by immunohistochemical staining in 115 pGBMs. VEGF protein expression was also assessed by immunohistochemical staining in 64 pGBM samples. Representative antibody stainings for Ki-67 with scale 0–3 are shown in Figure 2. Correlations of IDH1 mutation with Ki-67, MGMT, EGFR, VEGF, PTEN and mutant P53 expression status in pGBMs are analysed by two-sided χ^2^ test. As shown in Table 1, IDH1 mutation was associated with methylated MGMT promoter (*p* = 0.015), low expression of Ki-67 (*p* = 0.018) or EGFR (*p* = 0.012) and high level of mutant P53 proteins (*p* = 0.022), but not with MGMT, VEGF and PTEN expression levels.

### The independence of IDH1 mutation as a prognostic factor was limited in pGBM patients

We first conducted univariate cox regression analysis using clinical and genetic variables for the total 118 Chinese patients with pGBM, and found that age, preoperative KPS score, extent of resection, TMZ chemotherapy, Ki-67 protein expression level, and IDH1 mutation were statistically associated with PFS and OS, while sex and MGMT promoter methylation were not associated with PFS and OS (Table S1). Furthermore, the multivariable regression analysis and stepwise variable selection were then used to evaluate the independent prognostic value of these clinicopathologic factors on patient survival. The independence of IDH1 mutation as a prognostic factor was limited in 118 pGBM patients when considering gender, age, preoperative KPS score, extent of resection, TMZ chemotherapy, and Ki-67 protein expression level (HR, 0.62; 95%CI, 0.32–1.22; p = 0.17 for OS; HR, 0.62; 95%CI, 0.34–1.11; p = 0.11 for PFS; Table S1). Ki-67 is a more independent indicator than IDH1 mutation in our GBM samples. We also provided a multivariable cox model without Ki-67 to re-evaluate the association between IDH mutation and outcomes (Table S2). And as shown in Table S2, the P values of IDH1 mutation in Multivariate cox model were reduced to 0.09 (Overall Survival) and 0.05 (Progression-Free Survival) regardless of Ki-67 expression.

## Discussion

IDH1 mutations were initially discovered in a subset of GBMs by large-scale sequencing [11], and nearly 93% of IDH1 mutations are of the R132H variant. However, subsequent studies reported that IDH1 mutations were detected at much higher frequencies ranged from 60% to 80% in WHO grades II and III gliomas as well as in secondary GBMs. In contrast, it has been reported only 5 to 10% of pGBMs were accompanied with IDH1 mutations [12]. IDH1 mutations are an early event in tumorigenesis, and an independent favorable prognostic marker in human gliomas [13]. In the present study, IDH1 (IDH1R132) mutations existed in 16.1% pGBM samples among a large cohort of 118 Chinese patients. It is suspected that pGBM with IDH1 mutation may evolve from relative low grade glioma although without surgery history. In China, low grade gliomas offer a significantly higher percentage than that of Western countries. From the above, the high percentage of low grade gliomas in China may be the reason of higher frequency of IDH1 mutation in the Chinese pGBM population. Besides, 89.5% of IDH1 mutations were G395A transition (Arg→His, R132H), followed by C394A transversion (Arg→Ser, R132S; 10.5%). In accordance with previous studies, IDH1 mutations were more likely to occur in younger patients and also predicted a better clinical outcome in pGBMs at our institute.

Enhanced cellular proliferation is a fundamental feature of the growth of GBM [14]. The epidermal growth factor receptor (EGFR) is overexpressed and induces proliferation in multiple cancers including pGBM [15], [16]. And Ki-67 is a widely accepted marker for cell proliferation in daily pathologic practice [17], [18]. In the present study, we discovered that there was significant lower Ki-67 and EGFR protein expression in the pGBM samples with mutated IDH1 when compared with those with wild-type IDH1. Therefore, the correlation between IDH1 mutation and a better clinical outcome in pGBM patients may be associated with the low proliferation rate accompanying IDH1 mutation.

Previously, it has been demonstrated that most (80%) of the GBM samples with mutated IDH1 or IDH2 genes also had a mutation of p53 gene [19]. Consistent with this notion, in this study, we identified pGBM samples with IDH1 mutations showed a much higher expression level of mutant P53 protein. It is well known that p53 is a tumor suppressor in various cancers [20], and mutant p53 has been testified to promote the tumor growth [21]. Due to the better prognosis in the group with IDH1 mutation, it was interesting that mutant P53 protein was at a higher expression level in the samples with mutated IDH1. The inherent mechanism needs further investigation.

Accumulating evidences showed that epigenetic silencing of MGMT by promoter methylation and its association with improved survival in GBM patients treated with alkylating agents including Temozolomide [22], [23]. Recent studies also proved that IDH1 or IDH2 mutations predicted longer survival and response to temozolomide in low-grade gliomas [8]. However, the association of IDH1 mutation with MGMT promoter methylation or protein expression has not been systematically investigated in pGBMs. In our cases, the pGBM samples with mutated IDH1 showed a higher MGMT promoter methylation compared to those with wild-type IDH1. However, no correlation was identified between IDH1 mutation status and MGMT protein expression levels in pGBM samples. The oncogenic function and molecular pathway of IDH1/2 have not been fully understood yet. Nevertheless, the most recent studies further suggested that IDH1/2 mutations were associated with a distinct DNA hypermethylation phenotype in gliomas [24]. Therefore, it is possible that IDH1/2 mutations were involved in oncogenesis by the inactivation of tumor suppressor genes through promoter hypermethylation.

To date, few reports pointed out that IDH1 mutations were associated with tumor locations [7]. In our present study, for the first time to our knowledge, we found that the majority of pGBMs (84.2%) with IDH1 mutations were located in the frontal lobe, with a much higher percentage than those with wild-type IDH1 (36.4%). This phenomenon indicates that there might be a direct relationship between IDH1 mutations and tumor location. And the mechanisms underlying the above phenomenon remain to be validated and deciphered on more samples and researches.

Almost every research on IDH1 mutation commonly reported that IDH1 mutation was a strong prognostic indicator [5]–[8]. Multivariable regression analysis has shown that IDH1 mutation is an independent prognostic factor in anaplastic oligodendroglial tumors when referring to type of surgery, KPS, age, location, the central histology review diagnosis, endothelial abnormalities, necrosis, and the molecular factors such as 1p/19q loss, EGFR amplification, and MGMT promoter methylation [25]. Sanson et al. also indicated that IDH1 mutations predicted a better outcome in grade 2, grade 3, and grade 4 gliomas after adjustment for grade, age, MGMT status, genomic profile, and treatment in the multivariate regression model [13]. In our study, 118 pure pGBM samples were subjected to IDH1 mutation testing and used for the following survival analysis. And our study also kept in line with the previous studies in univariable regression model. Furthermore, we make initial selection of prognostic factors using univariable regression analysis, and only those with potential association with survival were included in our further multivariable regression model. Our multivariable regression analysis showed that the independence of IDH1 mutation as a prognostic factor was limited in 118 pGBM patients when considering age, preoperative KPS score, extent of resection, TMZ chemotherapy, and Ki-67 protein expression level. In our previous study, we reported that Ki-67 was a very strong prognostic indicator in our center [26]. And Ki-67 is an independent prognostic factor in the multivariable regression model including IDH1 mutation. When Ki-67 was removed from the multivariable regression model, the P values of IDH1 mutation in multivariate regression model were reduced to 0.095 (Overall Survival) and 0.071 (Progression-Free Survival). These findings pointed out that the better clinical outcome conferred by IDH1 mutations was the results of younger age, low expression of Ki-67 protein, or any other molecular alterations associated with IDH1 mutations. This might indicate the limitation of IDH1 mutation as an independent prognostic factor in Chinese patients with pGBM in the future.

In summary, our study confirms that IDH1 mutation is a strong prognostic biomarker for a favourable clinical outcome of Chinese patients with pGBM. Our data underscored the associations between IDH1 mutation status and younger age, methylated MGMT promoter, high expression of mutant P53 protein, low expression of Ki-67 or EGFR protein in pGBM samples. Most notably, we identified that IDH1 mutation mainly occurred in pGBM cases which were located in the frontal lobe. Furthermore, IDH1 mutation was not an independent prognostic indicator in the multivariable regression model, which might narrow its prognostic power in Chinese pGBM patients in the future. This raises questions regarding the capacity of this test as an objective and reproducible biomarker for customized treatment in individual cases.

## Supporting Information

Table S1 **Cox proportional hazard regression analyses of IDH1 mutation and clinicopathologic characteristics in relation to clinical outcome in 118 Chinese patients with pGBM.**
(DOC)

Table S2 **Multivariable proportional hazard regression analyses of IDH1 mutation and clinicopathologic characteristics without Ki-67 expression.**
(DOC)

## References

[1] Quick A, Patel D, Hadziahmetovic M, Chakravarti A, Mehta M (2010). Current therapeutic paradigms in glioblastoma.. Rev Recent Clin Trials.

[2] Yan W, Zhang W, Jiang T (2011). Oncogene addiction in gliomas: implications for molecular targeted therapy.. J Exp Clin Cancer Res.

[3] Weller M, Felsberg J, Hartmann C, Berger H, Steinbach JP (2009). Molecular predictors of progression-free and overall survival in patients with newly diagnosed glioblastoma: a prospective translational study of the German Glioma Network.. J Clin Oncol.

[4] Weller M, Stupp R, Reifenberger G, Brandes AA, van den Bent MJ (2010). MGMT promoter methylation in malignant gliomas: ready for personalized medicine?. Nat Rev Neurol.

[5] Yan H, Parsons DW, Jin G, McLendon R, Rasheed BA (2009). IDH1 and IDH2 mutations in gliomas.. N Engl J Med.

[6] von Deimling A, Korshunov A, Hartmann C (2011). The next generation of glioma biomarkers: MGMT methylation, BRAF fusions and IDH1 mutations.. Brain Pathol.

[7] Christensen BC, Smith AA, Zheng S, Koestler DC, Houseman EA (2011). DNA methylation, isocitrate dehydrogenase mutation, and survival in glioma.. J Natl Cancer Inst.

[8] Houillier C, Wang X, Kaloshi G, Mokhtari K, Guillevin R (2010). IDH1 or IDH2 mutations predict longer survival and response to temozolomide in low-grade gliomas.. Neurology.

[9] Scherer HJ (1940). Cerebral astrocytomas and their derivatives.. Am J Cancer.

[10] Zhang W, Qiu XG, Chen BS, Li SW, Cui Y (2009). Antiangiogenic therapy with bevacizumab in recurrent malignant gliomas: analysis of the response and core pathway aberrations.. Chin Med J (Engl).

[11] Parsons DW, Jones S, Zhang X, Lin JC, Leary RJ (2008). An integrated genomic analysis of human glioblastoma multiforme.. Science.

[12] Ichimura K, Pearson DM, Kocialkowski S, Bäcklund LM, Chan R (2009). IDH1 mutations are present in the majority of common adult gliomas but rare in primary glioblastomas.. Neuro Oncol.

[13] Sanson M, Marie Y, Paris S, Idbaih A, Laffaire J (2009). Isocitrate dehydrogenase 1 codon 132 mutation is an important prognostic biomarker in gliomas.. J Clin Oncol.

[14] Kanu OO, Mehta A, Di C, Lin N, Bortoff K (2009). Glioblastoma multiforme: a review of therapeutic targets.. Expert Opin Ther Targets.

[15] Herbst RS (2004). Review of epidermal growth factor receptor biology.. Int J Radiat Oncol Biol Phys.

[16] Ciardiello F, Tortora G (2008). EGFR antagonists in cancer treatment.. N Engl J Med.

[17] Katzenberger T, Petzoldt C, Höller S, Mäder U, Kalla J (2006). The Ki67 proliferation index is a quantitative indicator of clinical risk in mantle cell lymphoma.. Blood.

[18] Burcombe R, Wilson GD, Dowsett M, Khan I, Richman PI (2006). Evaluation of Ki-67 proliferation and apoptotic index before, during and after neoadjuvant chemotherapy for primary breast cancer.. Breast Cancer Res.

[19] Labussiere M, Sanson M, Idbaih A, Delattre JY (2010). IDH1 Gene Mutations: A New Paradigm in Glioma Prognosis and Therapy?. The Oncologist.

[20] Wang W, Rastinejad F, El-Deiry WS (2003). Restoring p53-dependent tumor suppression.. Cancer Biol Ther.

[21] Muller PA, Caswell PT, Doyle B, Iwanicki MP, Tan EH (2009). Mutant p53 drives invasion by promoting integrin recycling.. Cell.

[22] Hegi ME, Diserens AC, Gorlia T, Hamou MF, de Tribolet N (2005). MGMT gene silencing and benefit from temozolomide in glioblastoma.. N Engl J Med.

[23] Fukushima T, Takeshima H, Kataoka H (2009). Anti-glioma therapy with temozolomide and status of the DNA-repair gene MGMT.. Anticancer Res.

[24] Noushmehr H, Weisenberger DJ, Diefes K, Phillips HS, Pujara K (2010). Identification of a CpG island methylator phenotype that defines a distinct subgroup of glioma.. Cancer Cell.

[25] van den Bent MJ, Dubbink HJ, Marie Y, Brandes AA, Taphoorn MJ (2010). IDH1 and IDH2 mutations are prognostic but not predictive for outcome in anaplastic oligodendroglial tumors: a report of the European Organization for Research and Treatment of Cancer Brain Tumor Group.. Clin Cancer Res.

[26] Jin Q, Zhang W, Qiu XG, Yan W, You G (2011). Gene expression profiling reveals Ki-67 associated proliferation signature in human glioblastoma.. Chin Med J (Engl).

