# Supplementary material for: Correlation of IDH1 Mutation with Clinicopathologic Factors and Prognosis in Primary Glioblastoma: A Report of 118 Patients from China
Source: PLoS One. 2012 Jan 23;7(1):e30339. doi: 10.1371/journal.pone.0030339 (PMC3264567; doi:10.1371/journal.pone.0030339)
Supplement: Table S1 — Cox proportional hazard regression analyses of IDH1 mutation and clinicopathologic characteristics in relation to clinical outcome in 118 Chinese patients with pGBM. (DOC) [file pone.0030339.s001.doc]

**Table S1. Cox proportional hazard regression analyses of IDH1 mutation and clinicopathologic characteristics in relation to clinical outcome in 118 Chinese patients with pGBM**

| **Variable (number/ proportion)** | **Univariable Regression** | | | **Multivariable Regression** | | |
| --- | --- | --- | --- | --- | --- | --- |
|  | **HR** | **95% CI** | **p Value** | **HR** | **95% CI** | **p Value** |
| **Overall Survival** |  |  |  |  |  |  |
| Gender |  |  |  |  |  |  |
| Female (44/37.29%) | 1.00 |  |  | 1.00 |  |  |
| Male (74/62.71%) | 1.23 | 0.80-1.89 | 0.34 | 1.22 | 0.78-1.90 | 0.38 |
| Increasing age | 1.02 | 1.01-1.04 | 0.01 | 1.01 | 0.99-1.03 | 0.24 |
| KPS score |  |  |  |  |  |  |
| ≦80 (42/35.59%) | 1.00 |  |  | 1.00 |  |  |
| >80 (76/64.41%) | 0.31 | 0.20-0.49 | <0.01 | 0.44 | 0.25-0.78 | <0.01 |
| Extent of resection |  |  |  |  |  |  |
| Subtotal (48/40.68%) | 1.00 |  |  | 1.00 |  |  |
| Total (70/59.32%) | 0.46 | 0.30-0.71 | <0.01 | 0.85 | 0.49-1.49 | 0.58 |
| TMZ chemotherapy |  |  |  |  |  |  |
| No TMZ (85/72.03%) | 1.00 |  |  | 1.00 |  |  |
| TMZ (33/27.97%) | 0.51 | 0.31-0.84 | <0.01 | 0.48 | 0.28-0.82 | <0.01 |
| Ki-67 expression |  |  |  |  |  |  |
| Low Ki-67 (43/37.39%) | 1.00 |  |  | 1.00 |  |  |
| High Ki-67 (72/62.61%) | 1.64 | 1.23-2.17 | <0.01 | 1.56 | 1.12-2.17 | <0.01 |
| IDH1 mutation status |  |  |  |  |  |  |
| No mutation (99/83.9%) | 1.00 |  |  | 1.00 |  |  |
| Mutation (19/16.1%) | 0.50 | 0.27-0.94 | 0.03 | 0.62 | 0.32-1.22 | 0.17 |
| MGMT promoter methylation |  |  |  |  |  |  |
| No methylation (52/67.53%) | 1.00 |  |  |  |  |  |
| Methylation (25/32.47%) | 1.01 | 0.99-1.02 | 0.89 |  |  |  |
| **Progression-Free Survival** |  |  |  |  |  |  |
| Gender |  |  |  |  |  |  |
| Female (44/37.29%) | 1.00 |  |  | 1.00 |  |  |
| Male (74/62.71%) | 1.31 | 0.88-1.96 | 0.19 | 1.44 | 0.94-2.19 | 0.09 |
| Increasing age | 1.02 | 1.01-1.04 | 0.02 | 1.01 | 0.99-1.03 | 0.19 |
| KPS score |  |  |  |  |  |  |
| ≦80 (42/35.59%) | 1.00 |  |  | 1.00 |  |  |
| >80 (76/64.41%) | 0.44 | 0.29-0.67 | <0.01 | 0.56 | 0.34-0.93 | 0.56 |
| Extent of resection |  |  |  |  |  |  |
| Subtotal (48/40.68%) | 1.00 |  |  | 1.00 |  |  |
| Total (70/59.32%) | 0.60 | 0.40-0.89 | 0.01 | 1.01 | 0.61-1.67 | 0.97 |
| TMZ chemotherapy |  |  |  |  |  |  |
| No TMZ (85/72.03%) | 1.00 |  |  | 1.00 |  |  |
| TMZ (33/27.97%) | 0.59 | 0.35-0.93 | 0.02 | 0.51 | 0.31-0.84 | <0.01 |
| Ki-67 expression |  |  |  |  |  |  |
| Low Ki-67 (43/37.39%) | 1.00 |  |  | 1.00 |  |  |
| High Ki-67 (72/62.61%) | 1.59 | 1.23-2.07 | <0.01 | 1.59 | 1.19-2.13 | <0.01 |
| IDH1 mutation status |  |  |  |  |  |  |
| No mutation (99/83.9%) | 1.00 |  |  | 1.00 |  |  |
| Mutation (19/16.1%) | 0.54 | 0.31-0.94 | 0.03 | 0.62 | 0.34-1.11 | 0.11 |
| MGMT promoter methylation |  |  |  |  |  |  |
| No methylation (52/67.53%) | 1.00 |  |  |  |  |  |
| Methylation (25/32.47%) | 1.00 | 0.99-1.02 | 0.95 |  |  |  |
