# Supplementary material for: Correlation of IDH1 Mutation with Clinicopathologic Factors and Prognosis in Primary Glioblastoma: A Report of 118 Patients from China
Source: PLoS One. 2012 Jan 23;7(1):e30339. doi: 10.1371/journal.pone.0030339 (PMC3264567; doi:10.1371/journal.pone.0030339)
Supplement: Table S2 — Multivariable proportional hazard regression analyses of IDH1 mutation and clinicopathologic characteristics without Ki-67 expression. (DOC) [file pone.0030339.s002.doc]

**Table S2. Multivariable proportional hazard regression analyses of IDH1 mutation and clinicopathologic characteristics without Ki-67 expression**

| **Variable**  **(number/ proportion)** | **Multivariable Regression Analysis** | | |
| --- | --- | --- | --- |
|  | **HR** | **95% CI** | **p Value** |
| **Overall Survival** |  |  |  |
| IDH1 mutation status |  |  |  |
| No mutation (99/83.9%) | 1.00 |  |  |
| Mutation (19/16.1%) | 0.57 | 0.29-1.09 | 0.09 |
| Gender |  |  |  |
| Female (44/37.29%) | 1.00 |  |  |
| Male (74/62.71%) | 1.20 | 0.77-1.85 | 0.41 |
| Increasing age | 1.01 | 0.99-1.03 | 0.31 |
| KPS score |  |  |  |
| ≦80 (42/35.59%) | 1.00 |  |  |
| >80 (76/64.41%) | 0.44 | 0.25-0.79 | <0.01 |
| Extent of resection |  |  |  |
| Subtotal (48/40.68%) | 1.00 |  |  |
| Total (70/59.32%) | 0.72 | 0.41-1.26 | 0.24 |
| TMZ chemotherapy |  |  |  |
| No TMZ (85/72.03%) | 1.00 |  |  |
| TMZ (33/27.97%) | 0.51 | 0.31-0.85 | 0.01 |
| **Progression-Free Survival** |  |  |  |
| IDH1 mutation status |  |  |  |
| No mutation (99/83.9%) | 1.00 |  |  |
| Mutation (19/16.1%) | 0.56 | 0.32-1.00 | 0.05 |
| Gender |  |  |  |
| Female (44/37.29%) | 1.00 |  |  |
| Male (74/62.71%) | 1.40 | 0.93-2.13 | 0.11 |
| Increasing age | 1.01 | 0.99-1.03 | 0.24 |
| KPS score |  |  |  |
| ≦80 (42/35.59%) | 1.00 |  |  |
| >80 (76/64.41%) | 0.57 | 0.33-0.96 | 0.04 |
| Extent of resection |  |  |  |
| Subtotal (48/40.68%) | 1.00 |  |  |
| Total (70/59.32%) | 0.82 | 0.50-1.37 | 0.46 |
| TMZ chemotherapy |  |  |  |
| No TMZ (85/72.03%) | 1.00 |  |  |
| TMZ (33/27.97%) | 0.55 | 0.34-0.89 | 0.01 |
